# Revisiting the Evolution of Multi-Scale Structures of Starches with Different Crystalline Structures During Enzymatic Digestion

**DOI:** 10.3390/foods13203291

**Published:** 2024-10-17

**Authors:** Simin Chen, Zihui Qiu, Ying Yang, Jianfeng Wu, Wenjuan Jiao, Ying Chen, Chengzhi Jin

**Affiliations:** 1 Guangdong Provincial Key Laboratory of Molecular Target & Clinical Pharmacology, National Medical Products Administration, State Key Laboratory of Respiratory Disease, The Fifth Affiliated Hospital, School of Pharmaceutical Sciences, Guangzhou Medical University, Guangzhou 511436, China; sammiechan123@outlook.com; 2 College of Life Sciences, Fujian Normal University, Fuzhou 350117, China; ccdfafu@163.com (Z.Q.); yingyang1007@163.com (Y.Y.); 3 College of Food Science, South China Agricultural University, Guangzhou 510642, China; jianfeng.wu@scau.edu.cn; 4 Sericultural & Agri-Food Research Institute Guangdong Academy of Agricultural Sciences, Key Laboratory of Functional Foods, Ministry of Agriculture and Rural Affairs, Guangdong Key Laboratory of Agricultural Products Processing, Guangzhou 510610, China; jiaowenjuan@gdaas.cn; 5 School of Food Science and Engineering, Yangzhou University, Yangzhou 225127, China

**Keywords:** starch structures, enzymatic digestion, porous starch

## Abstract

Porous starch has been created through hydrolysis by amyloglucosidase and α-amylase. However, little information is known about the precise evolution of multi-scale structures of starch during digestion. In this study, rice starch and potato starch, containing different crystalline structures, were hydrolyzed by amyloglucosidase and α-amylase for 20 and 60 min, respectively, and their resulting structural changes were examined. The digestion process caused significant degradation of the molecular structures of rice and potato starches. In addition, the alterations in the ordered structures varied between the two starches. Rice starch exhibited porous structures, thicker crystalline lamellae as determined by small-angle X-ray scattering, and enhanced thermostability after digestion using differential scanning calorimetry. For rice starch, the extent of crystalline structures was analyzed with an X-ray diffractometer; it was found to first increase after 20 min of digestion and then decrease after 60 min of digestion. In contrast, potato starch did not display porous structures but exhibited thicker crystalline lamellae and a reduction in ordered structures after digestion. These findings suggest that it is possible to intentionally modulate the multi-scale structures of starch by controlling the digestion time, thereby providing valuable insights for the manipulation of starch functionalities.

## 1. Introduction

Starch holds great significance as a primary carbohydrate utilized in both food and non-food sectors. To meet the diverse requirements of various industries, starch granules are frequently subjected to modifications via physical, chemical, and enzymatic approaches [1,2]. Chemical modification, as a cost-effective and efficient method, has gained significant popularity as an industrial technique for altering starch functionalities [3]. However, concerns surrounding consumer health and environmental pollution have arisen due to the use of chemical treatments, thereby creating a pressing need for cleaner physical and enzymatic modification methods [3]. Physical modification, compared to chemical modification, offers higher consumer acceptance and safety while causing less harm to the environment. Nonetheless, it is often less efficient than chemical approaches and lacks the desired specificity to modify particular attributes of starch. In contrast, enzyme-based methods are structurally more specific, allowing for targeted alterations of starch structures, which play a crucial role in determining physicochemical properties. Therefore, enzymatic methods have gained wide acceptance for starch modification [2].

The use of α-amylase and amyloglucosidase enzymes for digesting starch granules has been widely employed in the modification of various starches, such as sweet potato starch, potato starch, wheat starch, and corn starch, to achieve desired changes in fine structures of starch and to create porous starch [4,5,6,7]. It has been observed that starches with different crystalline structures exhibited distinct digestion behaviors when subjected to enzymatic treatment [4,8,9,10]. Starches with an A-type crystalline structure generally follow an “inside-out” digestion pattern, whereas those with a B-type crystalline structure exhibit an “exo-pitting” digestion pattern [4,8,9,10]. Even though the digestion types of starches with different crystalline structures have been revealed, the evolution of multi-scale structures of starches during this digestion process has not been thoroughly understood yet. Additionally, the discrepancy in the structural changes in starches with different crystalline structures has not been discussed during digestion. It is crucial to unravel the precise changes in starch structures during enzymatic digestion because it holds the potential to enable the controllable manipulation of starch structures and functionalities by modulating digestion time. Thus, there is an urgent need to uncover the structural evolution of starches during digestion by α-amylase and amyloglucosidase.

Chen et al. [11] found that starch with an A-type crystalline structure displayed increased porosity during 20 min of digestion with α-amylase and amyloglucosidase. However, an extended digestion time of 120 min led to a significant breakdown of porous starch, proving counterproductive for porous starch preparation [11]. Accordingly, we believe that short-term digestion was enough for the preparation of porous starches. In this study, rice starch and potato starch, which, respectively, contained A- and B-type crystalline structures, were chosen as model starches with distinct crystalline structures to investigate the structural evolution of starches with different crystalline structures during enzymatic digestion. Hereby, short-term digestions (≤60 min) using α-amylase and amyloglucosidase were performed. Subsequently, various starch multi-scale structures, including morphology, lamellar structures, crystalline structures, ordered molecular structures, and fine structures, were meticulously investigated to uncover the evolutionary changes in starches with differing crystalline structures. These results provide valuable insights for modifying starch multi-scale structures and achieving desirable characteristics through simply controlling the digestion time.

## 2. Materials and Methods

### 2.1. Materials

Rice starch was purchased from Golden Agriculture Biotech Co., Ltd. (Wuxi, China). Potato starch was obtained from Foshan Gaofeng Starch Technology Co., Ltd. (Foshan, China). *Aspergillus niger* amyloglucosidase (A7095, 360 U/mL), porcine pancreatic *α*-amylase (A3176, 16 units/mg), and isoamylase (08124, ≥10,000,000 units/mg protein) were purchased from Sigma-Aldrich (Inc., St. Louis, MO, USA). All other reagents and chemicals used were of analytical grade.

### 2.2. Digestion of Starch Granules

In vitro digestibility of starches was determined using the Englyst method with minor modifications [11,12]. Briefly, 20 mL of sodium acetate buffer (0.1 mol/L, pH 5.2) was used to suspend starch (1 g, dry basis), followed by the addition of an enzyme solution (α-amylase: 1898 units; amyloglucosidase: 140 units) with 5 glass balls with a 3 mm diameter. Digestion was performed at 37 °C, 190 rpm. After digestion for 20 min or 60 min, starch suspensions were added to 30 mL of ethanol before centrifugation (2685× *g*, 10 min). The starch digesta were collected and washed with ethanol solution (60%, *v*/*v*) 3 times and then freeze-dried, ground, and sieved (150-mesh) for further analysis. The rice and potato starches digested for 20 and 60 min were referred to as Rice 20, Rice 60, Potato 20, and Potato 60, respectively.

### 2.3. Analysis of Granular Features of Starch Granules

Starch slurry (0.1 mg/mL) was prepared with distilled water, and the particle size of starch granules were analyzed using a Malvern MasterSizer 2000 (Malvern Instrument, Ltd., Malvern, UK). The specific surface area, D10, D50, and D90 were evaluated using Mastersizer 2000 software, where the D10, D50, and D90 values indicated the maximal particle size diameter that included 10%, 50%, and 90% of the particles (volume-weighted basis), respectively.

Additionally, a scanning electron microscope (SEM) (SU1510; Hitachi, Tokyo, Japan) was employed to observe the morphologies of starch granules. The samples were adhered and immobilized on conductive tape and then coated with gold in a vacuum evaporator and observed for their morphologies [13].

### 2.4. Analysis of Lamellar Structure of Starch Granules

The lamellar structure of starches was investigated by small-angle X-ray scattering (SAXS). Starch samples with a concentration of 60% (*w*/*w*) were prepared and equilibrated at 25 °C for 2 h prior to analysis. Each sample was placed into a paste cell subjected to X-rays for 5 min. Then, SAXS (SAXSpace, Anton-Paar, Graz, Austria) was applied for the analysis of the lamellar structure of starch granules, with the voltage set to 40 kV and a current set to 50 mA. The data were recorded using an image plate and collected by the IP Reader software (version 2023) using a phosphor imaging system (Cyclone Plus, PerkinElmer, New York, NY, USA).

To investigate changes in starch lamellae structures, including the thickness of semi-crystalline lamellae (*d*), amorphous lamellae (*d*_a_), and crystalline lamellae (*d*_c_), a one-dimension correlation profile was utilized based on the following equation, Equation (1) [14,15,16]:

(1) $$L\;\left(r\right)=\frac{\int_{0}^{\infty }I\left(q\right)q^{2}\cos\left(qr\right)dq}{\int_{0}^{\infty }I(q)q^{2}dq}$$

Here, *I* refers to the SAXS intensity, *r* (nm) represents the distance in real space, and *q* is the scattering vector. The thickness *d* was determined from the second maximum of *L*(*r*), *d*_a_ was obtained using the solution of the linear region and the flat minimum of *L*(*r*), and the thickness of the crystalline lamellae was calculated as *d*_c_ = *d* − *d*_a_.

### 2.5. Analysis of Crystalline Structure of Starches

The crystalline structure of starch was analyzed with an X-ray diffractometer (D8 Advance; Bruker, Madison, WI, USA) with a Cu-Kα X-ray source. The samples were scanned over a 2*θ* range of 5–50° at a scanning speed of 10°/min. The X-ray generator operated at 40 kV and 40 mA. Starch relative crystallinity (*RC*) was determined using MDI Jade 6.0 software, which calculated the ratio of the crystalline fraction to the total diffraction area [16].

### 2.6. Analysis of Ordered Molecular Structure of Starch Granules

A starch suspension with a concentration of 70% (*w*/*v*) was prepared by adding distilled water to a starch sample in an aluminum crucible of differential scanning calorimetry (DSC). The pans were sealed and equilibrated at 20 °C prior to the DSC analysis. Each sample was treated from 30 to 100 °C at a heating rate of 10 °C/min. An empty pan served as the reference. Gelatinization temperatures were recorded, including onset temperature (*T*_o_), peak temperature (*T*_p_), end temperature (*T*_e_), and enthalpy (∆*H*) [17].

### 2.7. Analysis of Degree of Branching of Starch Granules

About 5 mg of each starch was dissolved in 0.5 mL dimethyl sulfoxide (DMSO-*d*_6_) and heated in a water bath at 80 °C. ^1^H NMR spectra were measured with a Bruker 600 MHz (Bruker Corporation, Fallanden, Switzerland) at 30 °C. The acquisition parameters included a pulse angle of 30°, a delay time of 10 s, and an acquisition time of 2 s. Based on the peak assignment of ^1^H NMR spectra for starches [18], the anomeric signals for the α-1,4 linkage and α-1,6 linkage were observed at approximately ca. 5.1 ppm and ca. 4.9 ppm, respectively, and the peak at ca. 5.4 ppm was associated with the terminal non-reducing end linkage. The degree of branching (DB) was calculated using Equation (2) [18,19]:

(2) $$DB\left(\%\right)=\frac{I_{\alpha -1,6}}{I_{\alpha -1,4}+I_{\alpha -1,6}+I_{r-e}}\times 100$$

in which *I*_α-1,4_, *I*_α-1,6_, and *I*_r-e_ represent the peak area of α-1,4 linkage, α-1,6 linkage, and the terminal non-reducing end linkage, respectively.

### 2.8. Analysis of Molecular Structures of Starch Granules

Each starch sample (5 mg) was equilibrated with 5 mL of a dimethyl sulfoxide solution containing lithium bromide (0.5% *w*/*w*) (DMSO/LiBr), followed by heat treatment in a thermomixer at 80 °C for 3 h. Then, the molecular weight of the starches was determined using SEC-MALLS-RI, a size exclusion chromatography system combined with a differential detector and multi-angle laser light scattering. The average molecular weight (*M*_w_) of the starches in DMSO/LiBr (0.5% *w*/*w*) solution was examined with a DAWN HELEOS-II laser photometer (He-Ne laser, λ = 663.7 nm, Wyatt Technology Co., Santa Barbara, CA, USA). This system was equipped with three tandem columns (300 × 8 mm, Shodex OH-pak SB-805, 804 and 803; Showa Denko K.K., Tokyo, Japan) and maintained at 60 °C with a model column heater. With technical support from Sanshu Biotech Co., Ltd. (Shanghai, China), data were collected and analyzed using ASTRA6.1 software from Wyatt Technology [19].

To analyze changes in starch chain-length distributions during digestion, 5 mg of starch was blended with 0.9 mL of water and heated in a boiling water bath for 15 min. Then, the starch dispersion was added with 5 mL of sodium azide solution (40 mg/mL), 0.1 mL of acetate buffer (0.1 M, pH 3.5, prepared from 0.1 M acetic acid solution and 0.1 M sodium acetate solution), and 10 µL of isoamylase (1400 U). The mix was heated at 37 °C for 3 h and then precipitated with 5 mL of absolute ethanol. After centrifugation (4000× *g*, 10 min), the collected debranched starch was redissolved in 1 mL DMSO/LiBr solution and incubated at 80 °C, 350 rpm for 2 h. To determine the molecular weight distribution of debranched starch, samples were subjected to an Agilent 1260 Series SEC system equipped with a differential refractive index detector (Optilab T-rEX, Wyatt Technology Co., Santa Barbara, CA, USA) and two tandem columns (300 × 7.5 mm, Plael 10 µm MIXED-B and Plgel 5 µm MIXED-D; Agilent Technologies Inc., Santa Clara, CA, USA). The system was maintained at 80 °C with a model column heater, and the flow rate was set to 0.8 mL/min. The amylose content was calculated as the ratio of the area under the curve (AUC) of the debranched SEC distribution curves for the larger branches to the total AUC for all branches [20]. Technical support was provided by Sanshu Biotech Co., Ltd.

### 2.9. Statistical Analysis

All tests were performed at least in triplicate and statistical analyses were carried out using IBM SPSS statistics software, version 21.0 (IBM Corp., Armonk, NY, USA). Two-way analysis of variance (ANOVA) was applied with Tukey’s HSD tests (α = 0.05). The significance level was set as *p* < 0.05.

## 3. Results and Discussion

### 3.1. Morphology and Particle Size of Starches

**Figure 1:**
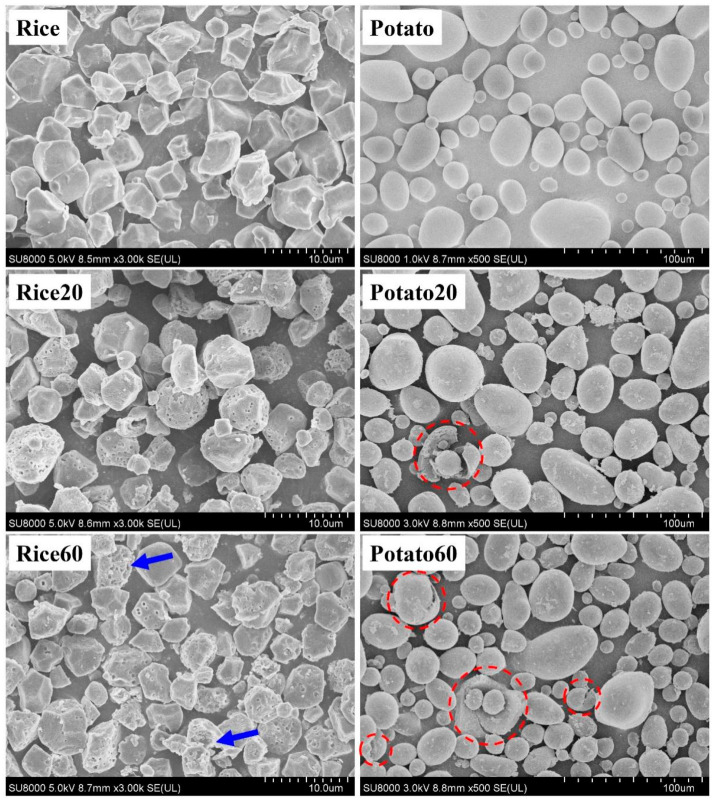
Morphology of starches. Rice/potato 20/60 refers to the corresponding starch after enzymatic digestion for 20/60 min. The red dotted circles indicate the breakdown of potato starch. The blue arrows indicate the starches that were greatly digested.

The morphology of starches is shown in Figure 1. Rice starch granules were characterized by small-sized particles (<10 μm) with an angular polyhedral appearance, whereas potato starch granules exhibited larger sizes (10–60 μm) with globular and ellipsoid shapes. After enzymatic digestion for 20 min, rice starch granules displayed pores on their surface, while potato starch granules exhibited rough structures with only a few cracks appearing. These observations confirmed that rice starch underwent enzymatic digestion following the “inside-out” pattern, while potato starch followed the “exo-pitting” pattern [4,8,9,10]. Furthermore, when the digestion time was extended to 60 min, greater digestion was observed on the surface of rice starch granules (as indicated by the blue arrows), but there was no significant breakdown of the particles. This indicated that a 60 min digestion period could be suitable for the preparation of porous rice starch. However, for potato starch, after 60 min of digestion, there were more rough structures and cracks without any pores, suggesting that enzymatic digestion could be not used for the preparation of porous potato starch.

**Figure 2:**
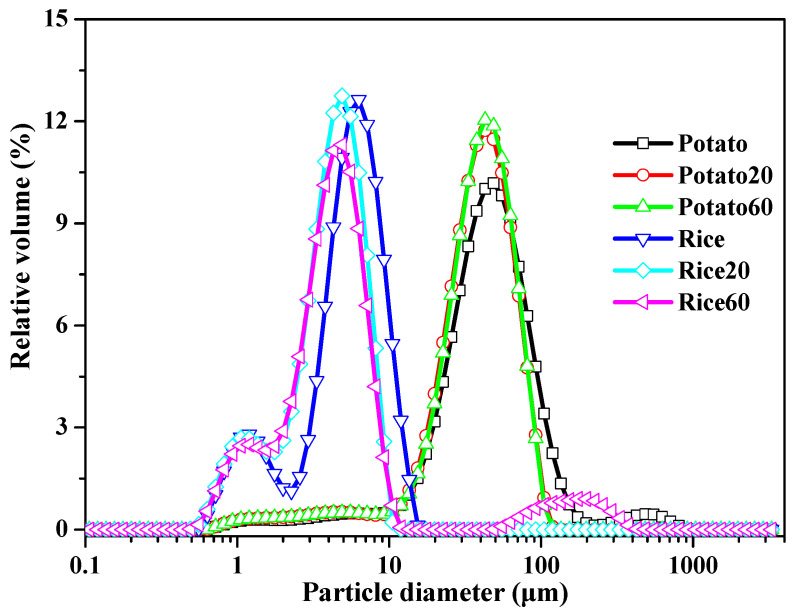
Size distribution of starches.

The particle size of starch granules is depicted in Figure 2 and Table S1. Rice starch exhibited major peaks in the range of approximately 0.6–15 μm, while potato starch showed peaks in the range of 10–200 μm. Following digestion, the specific surface area increased, and the D10, D50, and D90 values decreased with digestion time (Table S1), suggesting a decrease in starch granule size. These findings indicate that the enzymes effectively hydrolyzed the starch granules. However, the digestion time did not significantly affect the size of starch granules.

### 3.2. Lamellar Structures of Starches

**Figure 3:**
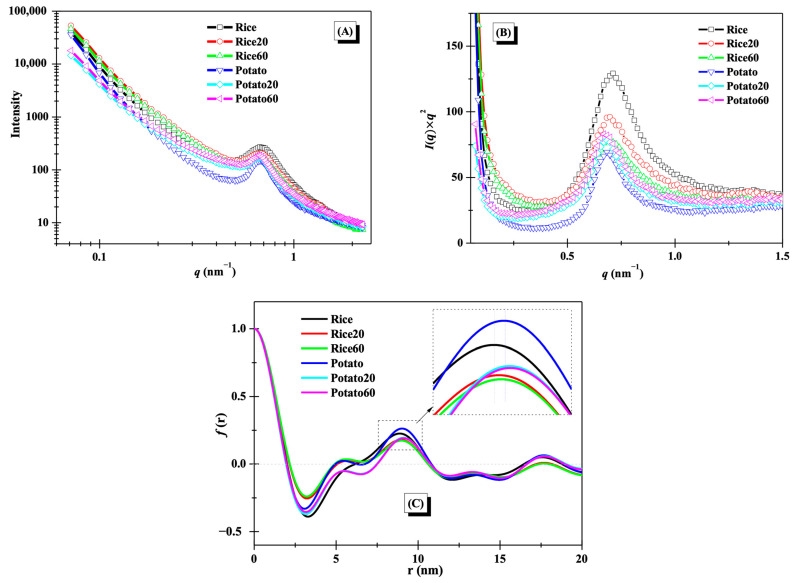
Lamellar structures of starches. (**A**), SAXS curves; (**B**), Kratky plots; (**C**) one-dimension correlation profiles.

The lamellar structures of starches were studied using small-angle X-ray scattering (SAXS), as illustrated in Figure 3A. A distinct peak at approximately 0.65 nm^−1^ was observed for the SAXS curves, suggesting the presence of lamellar structures [11]. After enzymatic digestion, notable changes were observed in the SAXS curves for both rice starch and potato starch, revealing significant alterations to starch lamellar structures. To gain further insights into these changes, Kratky plots (*I*(*q*) × *q*^2^ vs. *q*) are presented in Figure 3B. The peak area and position of both rice starch and potato starch showed noticeable modifications during digestion, reflecting the disorganization of lamellar structures and changes in lamellar thickness [21]. For rice starch, the peak area was decreased with increasing digestion time, while it showed an opposite trend for potato starch.

One-dimension correlation profiles as shown in Figure 3C were used to clearly evaluate the changes in the thickness of lamellar structures. The thickness of semi-crystalline lamellae (*d*), amorphous lamellae (*d*_a_), and crystalline lamellae (*d*_c_) were calculated based on Equation (1) in Section 2.4 and are summarized in Table 1. The *d* and *d*_c_ values of both rice starch and potato starch increased after digestion. The change in the *d* value was consistent with previous findings that the *d* of maize starch increased during 20–120 min of digestion [11]. The increase in *d* was probably due to the digestion of amorphous structures and the following swelling of crystalline lamellae [21]. However, the *d*_a_ value of rice starch significantly decreased, while that of potato starch significantly increased. This difference was attributed to the variations in crystalline structures between the two starches. Notably, the digestion time did not significantly affect the thickness of semi-crystalline lamellae, amorphous lamellae, or crystalline lamellae (Table 1), in line with the observation of an overlapping curve between Rice20 and Rice60 or between Potato20 and Potato60 in Figure 3C. This phenomenon was probably due to the limited digestion duration (≤60 min) used in this study. Chen et al. [11] found that longer digestion time (120 min) remarkably affected the thickness of starch lamellar structures.

### 3.3. Crystalline Structures of Starches

**Figure 4:**
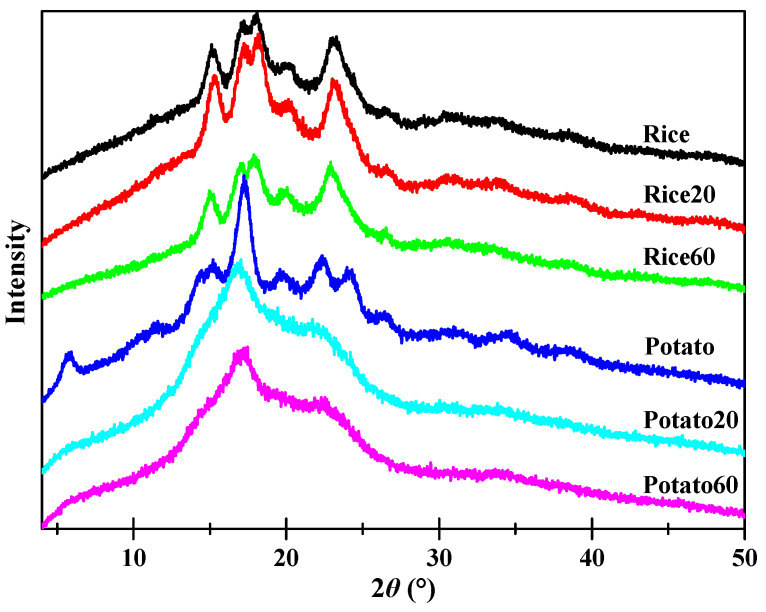
X-ray diffraction patterns of starches.

X-ray diffraction patterns of both native and digested starches are shown in Figure 4. All rice starch granules displayed prominent peaks at approximately 15.1, 17.0, 18.0, and 23.1° (2*θ*), suggestive of the presence of an A-type crystalline structure [22]. On the other hand, potato starches exhibited peaks at approximately 5.7, 15.2, 17.0, 22.2, and 23.1° (2*θ*), suggesting the existence of a B-type crystalline structure [11]. Enzymatic digestion did significantly affect the intensity of the peaks for both rice starch and potato starch, indicating variations in their crystalline structures. Rice20 showed higher peak intensities than Rice, which could be attributed to the digestion of amorphous structures and, subsequently, an increase in the relative content of crystalline structures. With prolonged digestion time, Rice60 exhibited weaker peak intensities compared to Rice20, confirming that extended digestion also contributed to the disorganization of crystalline structures. As for potato starch, digestion led to a decrease in peak intensity over time, implying that the digestion process contributes to the disorganization of crystalline structures.

Table 1 shows the relative crystallinity (*RC*) of starches. The *RC* of rice starch initially increased but then decreased, while it continuously decreased for potato starch, as the digestion time increased from 20 to 60 min. Enzymes are known to have a higher susceptibility towards amorphous structures compared to ordered structures [23]. Consequently, during enzymatic digestion, amorphous structures are typically targeted first. In the case of rice starch granules, digestion proceeded in an “inside-out” pattern, with the interior amorphous structures being easily digested. As a result, rice starch exhibited higher crystallinity after digestion for 20 min. However, as the digestion time increased, enzymes also attacked crystalline structures, leading to a lower crystallinity in Rice60 compared to Rice20. As for potato starch, digestion occurred in an “exo-pitting” pattern, indicating that both amorphous and crystalline structures were susceptible to digestion. Consequently, the crystalline structure of potato starch became disorganized along with digestion.

### 3.4. Ordered Molecular Structures of Starches

DSC thermograms and thermal properties of starches are shown in Figure S1 and Table 2, respectively. During the digestion process, both rice starch and potato starch exhibited a decrease in enthalpy, indicating a disorganization of their structures. For rice starch, digestion led to an increase in the thermal properties (*T*_o_, *T*_p_, and *T*_e_) independent of digestion time, suggesting improved starch thermostability. This can be attributed to the susceptibility of amorphous and less-ordered structures to enzyme digestion, resulting in their removal [23]. Additionally, as starch granules undergo digestion, the remaining structures can reassemble and form ordered structures, thereby further enhancing their thermal properties [24]. In contrast, for potato starch, digestion caused a decrease in *T*_o_ and *T*_p_. This phenomenon could be explained by the fact that potato starch was digested in an “exo-pitting” pattern, with ordered structures close to the granular surface being disrupted and disorganized. With prolonged digestion time, a greater extent of digestion of less-ordered structures occurred, leading to structural rearrangements within the potato starch. This accounts for the observation of an increase in *T*_o_, while *T*_p_ and *T*_e_ remained unchanged. These findings highlight the significant impact of the crystalline structures of starch granules on the evolution of ordered molecular structures during digestion.

### 3.5. Degree of Branching of Starches

The ^1^H NMR spectra of starches are depicted in Figure S2, and the degree of branching (DB) was subsequently calculated and is presented in Table 2. The DB value of both rice starch and potato starch increased as the digestion time progressed, indicating a higher degradation of α-1,4-glucosidic linkages compared to α-1,6-glucosidic linkages. This can be ascribed to the specific hydrolysis of α-1,4-glucosidic linkages by α-amylase, while amyloglucosidase was capable of hydrolyzing both α-1,4-glucosidic and α-1,6-glucosidic linkages. Consequently, during digestion, there was a more pronounced hydrolysis of the α-1,4-glucosidic linkages in the starches since both α-amylase and amyloglucosidase can target these specific linkages.

### 3.6. Chromatographic Analysis of the Molecular Weight

**Figure 5:**
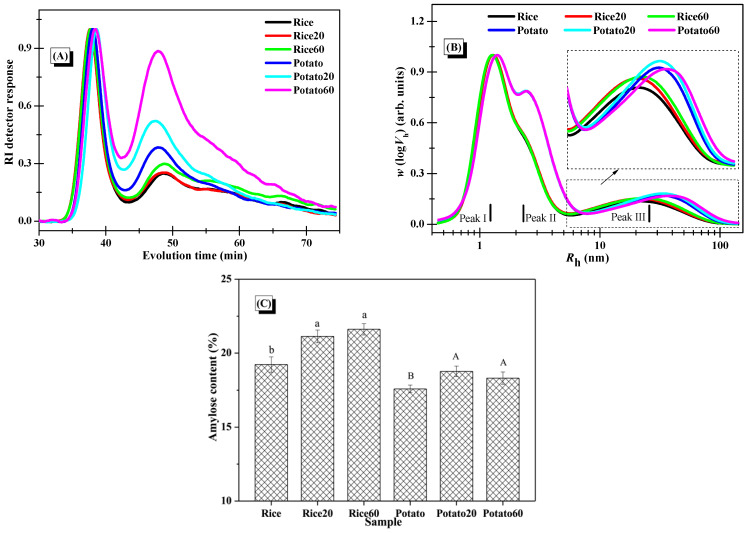
Changes in the molecular structures of starches. (**A**), Normalized RI response of the SEC-RI chromatogram of whole starches (normalization was carried out using the individual RI signal of the first eluted peak at around 34–43 min as the normalization factor for each sample); (**B**), SEC weight chain-length distributions of debranched starches with normalized *R_h_* signals (normalization was performed using the individual signal at Peak I as the normalization factor for each sample); (**C**), amylose content of starches (columns followed by different letters indicate the data differed significantly in digestion time within the same starch (*p <* 0.05)).

The average molar mass and distribution of starches were analyzed using SEC-RI, with the RI response displayed in Figure 5A. The findings demonstrated a common peak at around 34–43 min for all starch samples, corresponding to the elution of amylopectin. Furthermore, signals observed between 43 and 70 min indicated the elution of small amounts of amylopectin and amylose [25]. Post-digestion, both rice starch and potato starch exhibited a higher intensity of the peak at 43–70 min, indicating that the hydrolysis extent kept increasing along with digestion proceeding. This confirmed that both types of starch were significantly degraded during the digestion process. The changes in *M*_w_ of the starches, as summarized in Table 2, further support that the degradation of starch structures occurred as a function of digestion time.

### 3.7. Molecular Distribution of Starches

The distributions of debranched starches are depicted in Figure 5B. Peak I, Peak II, and Peak III were assigned to the short amylopectin branches, long amylopectin branches, and amylose fractions, respectively [25]. Upon digestion, the digested starch fractions, comprising both amylose and amylopectin, could be solubilized in the solution, and certain fractions could be separated and removed by centrifugation during the preparation of digested starch granules. Notably, there were no significant changes observed in Peak I and Peak II for both starch samples after the digestion, but there was an increase in the relative intensity of Peak III. This indicated greater changes in amylose structures. The amylose chains, which are dispersed within the amylopectin clusters, were positioned towards the outer surface of the granules, serving as reinforcing rods [26]. Accordingly, the presence of amylopectin in the digested starch might provide some protection for the digested amylose fractions, making it challenging for them to dissolve in the solution and be eliminated during the preparation of digested starch granules. As certain digested amylopectin fractions were removed during the starch preparation process, the fine structures of amylopectin in the collected starch digesta could remain largely unchanged in comparison to the native starch. Additionally, when comparing rice starch to potato starch, results showed that enzymatic digestion has similar effects on their starch fine structures, revealing no significant difference.

As presented in Figure 5C, enzymatic digestion significantly increased the amylose content in both starches, resulting from the removal of digested amylopectin during the preparation of starch digesta. Interestingly, the digestion time had no marked effect on the amylose content, possibly because both digested amylose and amylopectin were simultaneously removed during long-term digestion and the centrifugation process.

### 3.8. Short Discussion

Rice starch displayed an A-type crystalline structure, whereas potato starch exhibited a B-type crystalline structure. In the A-type polymorphic starch, α-1,6 branch linkages were distributed throughout both the crystalline and amorphous regions, whereas in the B-type starch, most of the branch linkages were concentrated in the amorphous region [27]. Wang et al. [28] further explained that the crystallite “weak points” of A-type starch were attributed to the larger number of short A-chains (degree of polymerization 6–12) derived from branch linkages located within the crystalline region compared to its B-type counterpart. Consequently, the crystalline lamellae of A-type starch contain “weak points”, making the interior of the starch more accessible to enzymes [27,28]. During the digestion process with α-amylase and amyloglucosidase, both the amorphous and crystalline regions of rice starch were susceptible to enzymatic digestion due to the presence of these “weak points”. However, the crystalline lamellae of potato starch were demonstrated with high resistance to enzyme digestion due to their well-defined, ordered structures, which efficiently hindered enzymatic action [23,29]. These differences in structural evolution between both starches during enzymatic digestion largely accounted for the contrasting digestion profiles observed.

Our findings confirmed that rice starch can be utilized to produce porous starch after undergoing digestion for 20 and 60 min, while potato starch was not suitable for the preparation of porous starch. However, it is worth noting that different applied digestion conditions could lead to different conclusions.

**Figure 6:**
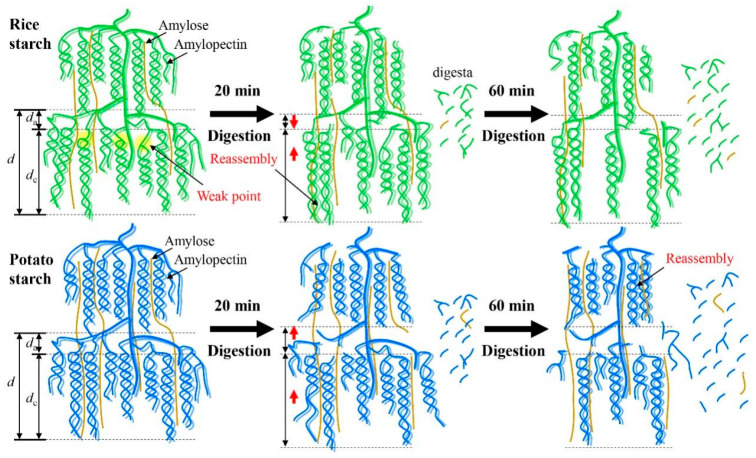
Schematic illustration of the changes in multi-scale structures of starches during enzymatic digestion. Rice starch is represented in green, while potato starch is shown in blue.

A previous study demonstrated that a defined porous structure was observed in the potato starch after the combination of extended acid hydrolysis and α-amylase or glucoamylase digestion [30]. The digestion process, driven by α-amylase and amyloglucosidase, exhibited substantial effects on modulating the fine structures and multi-scale ordered structures of starch. However, the digestion duration did not affect the size of starch granules probably due to the limited digestion time (up to 60 min) used in this study. In a previous work, it was observed that a longer digestion time (120 min) significantly decreased starch granular size [11]. Figure 6 provides a schematic illustration depicting the changes in multi-scale structures during enzymatic digestion. After digestion for 20 min, both the crystalline and amorphous lamellae of rice starch were hydrolyzed due to the presence of “weak points” within the crystalline lamellae. As a result, degradation of both amylose and amylopectin occurred, accompanied by the swelling of crystalline lamellae (Table 1). Notably, the digestion process resulted in notable reassembly of rice starch structures, as indicated by the observed changes in the *T*_o_ value. This reassembly may contribute to reducing the *d*_a_ value and increasing the presence of crystalline structures compared to the original starch (Table 1). However, with the prolongation of the digestion time up to 60 min, rice starch underwent further hydrolysis, leading to a significant disorganization of its ordered structures, while the *d*_a_ value remained unchanged (Table 1 and Table 2 and Figure 3). In the case of potato starch, digestion continually degraded its structures and disorganized its ordered structures as increasing digestion time (Table 1 and Table 2 and Figure 3). Additionally, swelling of the crystalline lamellae (Table 1) and slight reassembly of ordered structures (Table 2) were observed. Notably, potato starch experienced an increase in *d*_a_. This can be attributed to the digestion of crystalline lamellae in potato starch and the disruption of the well-defined helical structures. These findings strongly support the idea that starches with varying crystalline structures could undergo distinct structural changes during enzymatic digestion.

Porous starch has commonly been utilized as a carrier to deliver bioactive substances in the gastrointestinal tract [31]. Previous findings have established that the compactness of starch-based delivery systems heavily influenced the behavior of bioactive element delivery [32,33,34]. Following enzymatic digestion, rice starch exhibited porous structures, and the content of ordered structures could either increase or decrease depending on the duration of digestion. This suggests that porous rice starch could be considered a potential oral delivery system, and its delivery properties could be modified by controlling the content of ordered structures through adjusting digestion duration. Additionally, the digestion process tended to enhance the amylose content of both rice starch and potato starch. This increase in amylose content could greatly enhance the functionality of starch-based foods [35,36,37]. Overall, the digestion process, facilitated by amyloglucosidase and α-amylase, had a profound effect on the multi-scale structures of starch. The samples collected in this study could represent the discrepancy in the structural evolution of starch with different crystalline structures. However, further investigation would be required to determine the optimal digestion time for the modification of rice and potato starch and to fully understand how the changes in starch multi-scale structures influence variations in starch functionality. Moreover, it would be necessary to further investigate whether these findings hold true for other starches, such as wheat starch, pea starch, and cassava starch. Further research and analysis are warranted to establish the consistency of these findings across different starch types.

## 4. Conclusions

In this study, the changes in multi-scale structures of rice starch and potato starch during digestion by amyloglucosidase and α-amylase were examined. The digestion process resulted in significant degradation of starch molecular structures and a potential increase in amylose content for both starches. However, the changes in ordered structures varied between these two starches due to differences in their crystalline structures. After digestion, rice starch exhibited porous structures, thicker crystalline lamellae, and higher thermostability, with the crystalline structures either decreasing or increasing depending on the digestion time. On the other hand, potato starch did not display porous structures but showed increased thickness in the crystalline lamellae and a reduction in ordered structures. Overall, the digestion process had a profound impact on the multi-scale structures of starch. This study offers a promising approach to tailoring starch structures by controlling the digestion time, enabling the rational modulation of starch functionalities.

## Figures and Tables

**Table 1:** Thickness of lamellar structures and relative crystallinity (*RC*) of starches.

Sample	*d* (nm)	*d*_a_ (nm)	*d*_c_ (nm)	*RC* (%)
Rice	8.88 ± 0.01 ^b^	2.58 ± 0.01 ^a^	6.30 ± 0.01 ^b^	18.87 ± 0.47 ^b^
Rice20	8.95 ± 0.02 ^a^	2.53 ± 0.01 ^b^	6.42 ± 0.02 ^a^	19.80 ± 0.36 ^a^
Rice60	8.98 ± 0.01 ^a^	2.52 ± 0.01 ^b^	6.46 ± 0.01 ^a^	17.43 ± 0.21 ^c^
Potato	9.02 ± 0.01 ^B^	2.44 ± 0.01 ^B^	6.58 ± 0.01 ^B^	18.97 ± 0.25 ^A^
Potato20	9.11 ± 0.01 ^A^	2.48 ± 0.01 ^A^	6.63 ± 0.01 ^A^	16.23 ± 0.21 ^B^
Potato60	9.12 ± 0.02 ^A^	2.48 ± 0.01 ^A^	6.63 ± 0.02 ^A^	15.27 ± 0.15 ^C^

Note: Values are represented as mean ± SD *(n* = 3). Values followed by different uppercase or lowercase letters within a column differ significantly (*p* < 0.05) in digestion time within the same starch.

**Table 2:** Thermal properties, molecular weight (*M*_w_), and degree of branching (DB) of starches.

Sample	*T*_o_ (°C)	*T*_p_ (°C)	*T*_e_ (°C)	△H (J/g)	DB (%)	*M*_w_ (×10^6^ Da)
Rice	63.68 ± 0.09 ^b^	68.61 ± 0.42 ^b^	75.67 ± 0.35 ^b^	9.15 ± 0.94 ^a^	6.64 ± 0.15 ^c^	163.10 ± 0.52 ^a^
Rice20	68.44 ± 0.10 ^a^	72.83 ± 0.17 ^a^	81.20 ± 0.15 ^a^	5.53 ± 0.22 ^b^	7.27 ± 0.12 ^b^	161.26 ± 0.50 ^b^
Rice60	68.81 ± 0.20 ^a^	74.25 ± 1.53 ^a^	81.83 ± 1.75 ^a^	5.95 ± 0.25 ^b^	7.76 ± 0.13 ^a^	125.65 ± 0.38 ^c^
Potato	61.82 ± 0.02 ^A^	65.78 ± 0.09 ^A^	71.69 ± 0.62 ^A^	13.37 ± 0.89 ^A^	5.43 ± 0.09 ^C^	91.30 ± 0.23 ^A^
Potato20	55.82 ± 0.33 ^C^	62.17 ± 0.42 ^B^	71.80 ± 0.36 ^A^	10.30 ± 0.48 ^B^	5.74 ± 0.13 ^B^	59.75 ± 0.12 ^B^
Potato60	57.41 ± 0.29 ^B^	62.34 ± 0.23 ^B^	71.16 ± 0.04 ^A^	5.45 ± 0.26 ^C^	5.96 ± 0.01 ^A^	52.11 ± 0.10 ^C^

Note: Values are the mean ± SD (*n* = 3). Values followed by different uppercase or lowercase letters within a column differ significantly in digestion time within the same starch (*p <* 0.05).

## Data Availability

The data presented in this study are available on request from the corresponding authors due to privacy restrictions.

## Supplementary Materials

The following supporting information can be downloaded at: https://www.mdpi.com/article/10.3390/foods13203291/s1, Figure S1: DSC thermograms of starches, Figure S2: ^1^H NMR spectra of starches, Table S1: Particle diameter of starches.
