# Supplementary material for: Revisiting the Evolution of Multi-Scale Structures of Starches with Different Crystalline Structures During Enzymatic Digestion
Source: Foods. 2024 Oct 17;13(20):3291. doi: 10.3390/foods13203291 (PMC11507109; doi:10.3390/foods13203291)
Supplement: Supplementary file 1 [file foods-13-03291-s001.zip › foods-3135614-supplementary.pdf]

# Revisiting the elution of multi-scale structures of starches with different crystalline structures during the enzymatic digestion

Simin Chen [a], Zihui Qiu [b], Ying Yang [b], Jianfeng Wu [c], Wenjuan Jiao [d], Ying Chen [e, *], Chengzhi Jin [a, *]

[a] Guangdong Provincial Key Laboratory of Molecular Target & Clinical Pharmacology, National Medical Products Administration, State Key Laboratory of Respiratory Disease, The Fifth Affiliated Hospital, School of Pharmaceutical Sciences, Guangzhou Medical University, Guangzhou 511436, China

[b] College of Life Sciences, Fujian Normal University, Fuzhou 350117, China

[c] College of Food Science, South China Agricultural University, Guangzhou, China

[d] Sericultural & Agri-Food Research Institute, Guangdong Academy of Agricultural Sciences, Key Laboratory of Functional Foods, Ministry of Agriculture and Rural Affairs, Guangdong Key Laboratory of Agricultural Products Processing, Guangzhou, 510610, China

[e] School of Food Science and Engineering, Yangzhou University, Yangzhou 225127, China

[*] Corresponding author: Ying Chen and Chengzhi Jin

Email address: 008265@yzu.edu.cn (Y. Chen) and chengzhijin@gzhmu.edu.cn (C. Jin);

**Table S1** Particle diameter of starches

| Sample | Specific surface area ($m^2$/kg) | D10 (μm) | D50 (μm) | D90 (μm) |
|---|---|---|---|---|
| Rice | $184.32 \pm 4.54^{d}$ | $17.82 \pm 0.29^{a}$ | $45.32 \pm 0.89^{a}$ | $98.58 \pm 7.98^{a}$ |
| Rice20 | $211.90 \pm 6.11^{c}$ | $16.40 \pm 0.50^{b}$ | $39.26 \pm 0.71^{b}$ | $70.24 \pm 1.00^{b}$ |
| Rice60 | $209.98 \pm 6.39^{c}$ | $16.68 \pm 0.52^{b}$ | $39.86 \pm 0.15^{b}$ | $70.46 \pm 0.47^{b}$ |
| Potato | $1160.20 \pm 3.56^{b}$ | $1.34 \pm 0.00^{c}$ | $5.48 \pm 0.02^{c}$ | $9.48 \pm 0.06^{c}$ |
| Potato20 | $1374.00 \pm 0.71^{a}$ | $1.30 \pm 0.00^{c}$ | $4.20 \pm 0.00^{d}$ | $7.17 \pm 0.01^{d}$ |
| Potato60 | $1390.80 \pm 52.49^{a}$ | $1.31 \pm 0.04^{c}$ | $4.07 \pm 0.11^{d}$ | $7.28 \pm 0.80^{d}$ |

Note: Values were the mean ± SD ($n$ = 5). Values followed by different lowercase letters within a column differed significantly ($P < 0.05$).

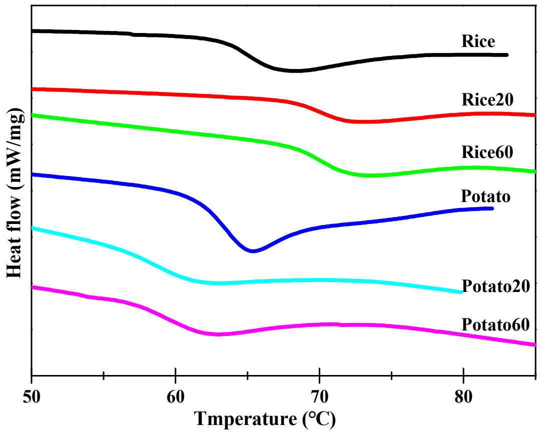


**Figure S1** DSC thermograms of starches

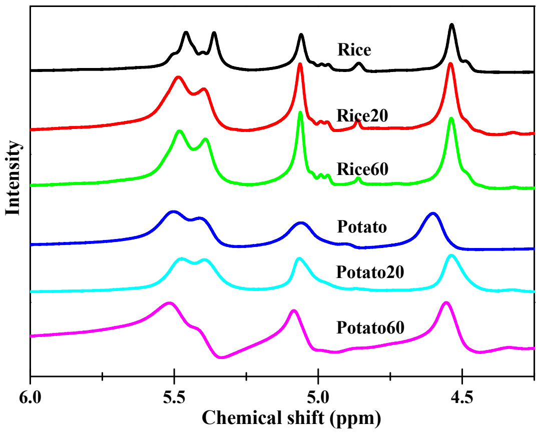


**Figure S2** $^{1}H$ NMR spectra of starches
